# The effect of glycerol as a cryoprotective agent in the cryopreservation of adipose tissue

**DOI:** 10.1186/s13287-022-02817-z

**Published:** 2022-04-08

**Authors:** Pei-Qi Zhang, Poh-Ching Tan, Yi-Ming Gao, Xiao-Jie Zhang, Yun Xie, Dan-Ning Zheng, Shuang-Bai Zhou, Qing-Feng Li

**Affiliations:** 1 Department of Plastic and Reconstructive Surgery, Shanghai Ninth People’s Hospital, Shanghai Jiao Tong University School of Medicine, 639 Zhizaoju Road, Shanghai, 200011 People’s Republic of China; 2 College of Life Sciences, Shanghai Normal University, Shanghai, People’s Republic of China

**Keywords:** Adipose tissue, Cryopreservation, Cryoprotective agent, Glycerol

## Abstract

**Background:**

Long-term preservation of adipose tissue is crucial for clinical applications. Researchers should consider both efficiency and biosafety when choosing a cryoprotective agent (CPA) for adipose tissue preservation. Glycerol has been applied as a nontoxic CPA for multiple tissues but not adipose tissue. We aimed to evaluate the efficacy of glycerol as a CPA for adipose tissue cryopreservation.

**Methods:**

Fresh human adipose tissues were obtained from patients who underwent liposuction and divided into 1 mL samples. Each sample was randomly mixed with 1 mL of CPA: 60–100% glycerol, 0.25 mol/L trehalose or DMSO + FBS and cryopreserved in − 196 °C liquid nitrogen for one month. After thawing and elution, the tissues were immediately evaluated for activity and structural integrity in vitro. Then, 0.2 mL of each sample was transplanted subdermally to the nude mouse dorsum and harvested after one month for histological examination to assess the effect of the cryopreserved fat in transplantation.

**Results:**

After cryopreservation, the samples treated with DMSO + FBS, trehalose, 60% and 70% glycerol had a more integrated structure than the samples in other groups. Tissues preserved with 70% glycerol had the highest G3PDH activity of 24.41 ± 0.70, comparable to 24.76 ± 0.48 in fresh tissue (*p* > 0.05). Adipose-derived stem cells (ASC) viability, proliferation and differentiation capability were also better preserved in 70% glycerol group. In vivo analysis showed that tissue preserved with 70% glycerol had a retention rate of 52.37 ± 7.53%, significantly higher than other groups. Histological observation demonstrated better structural integrity and viability in 70% glycerol group. Compared to the DMSO + FBS and trehalose groups, the glycerol groups showed lower tissue inflammation.

**Conclusion:**

Glycerol (70%) is efficient in adipose tissue cryopreservation. Glycerol-based CPAs, which are nontoxic and show biosafety, are a promising solution for clinical tissue cryopreservation.

**Supplementary Information:**

The online version contains supplementary material available at 10.1186/s13287-022-02817-z.

## Background

Autologous fat transplantation is considered an ideal method for soft tissue surgery and repair in plastic and reconstructive surgery due to its bioavailability and biocompatibility [1]. Fat grafting for reconstruction or aesthetic surgery usually requires multiple transplantations to achieve the treatment goals due to its unpredictable absorption rates [2]. Preserving adipose tissue after a single liposuction enables multiple reinjections at the proper time, thus eliminating repeated liposuction, increasing patient acceptance, and reducing costs and suffering. Moreover, adipose tissue contains many adipose-derived stem cells (ASCs), which have multipotent mesenchymal differentiation potential [3]. Addressing the problem of long-term preservation of adipose tissue can also provide a new solution for stem cell storage.

Cryopreservation has been studied both clinically [4, 5] and experimentally [6, 7] as a solution for tissue and cell preservation. Cryoprotective agents (CPAs) are necessary to reduce the damage caused by ice crystals formed during freezing [8]. Adipose tissue, as a composite tissue, requires CPAs that can effectively preserve multiple cell types, especially adipocytes containing large oil drops. However, in previous studies, researchers often applied traditional cellular CPAs that were either cytotoxic [9] or lacked evidence of efficacy [10].

Glycerol is a molecule that protects cells from freezing injury mainly by reducing intracellular ice crystal formation and osmotic pressure differences [11]. This molecule has been studied as a CPA for composite tissues such as testicular tissue [11], ovarian tissue [12], bones and cartilage [13]. As the backbone of triglycerides contained in adipocytes [14], glycerol has a potential advantage in adipose tissue cryopreservation.

Therefore, in this study, we sought to evaluate the feasibility and efficacy of glycerol-based CPAs for adipose tissue cryopreservation, thus looking for a safe and reliable CPA for future clinical use.

## Methods

### Harvest and preparation of adipose tissue

Human adipose tissues were obtained from discarded tissue from 5 healthy female patients (aged 21–46 years) who underwent abdominal liposuction. Written informed consent was obtained from all patients. This study was approved by the Ethics Committee of Shanghai Ninth People's Hospital and complied with the principles of the Declaration of Helsinki.

**Fig. 1:**
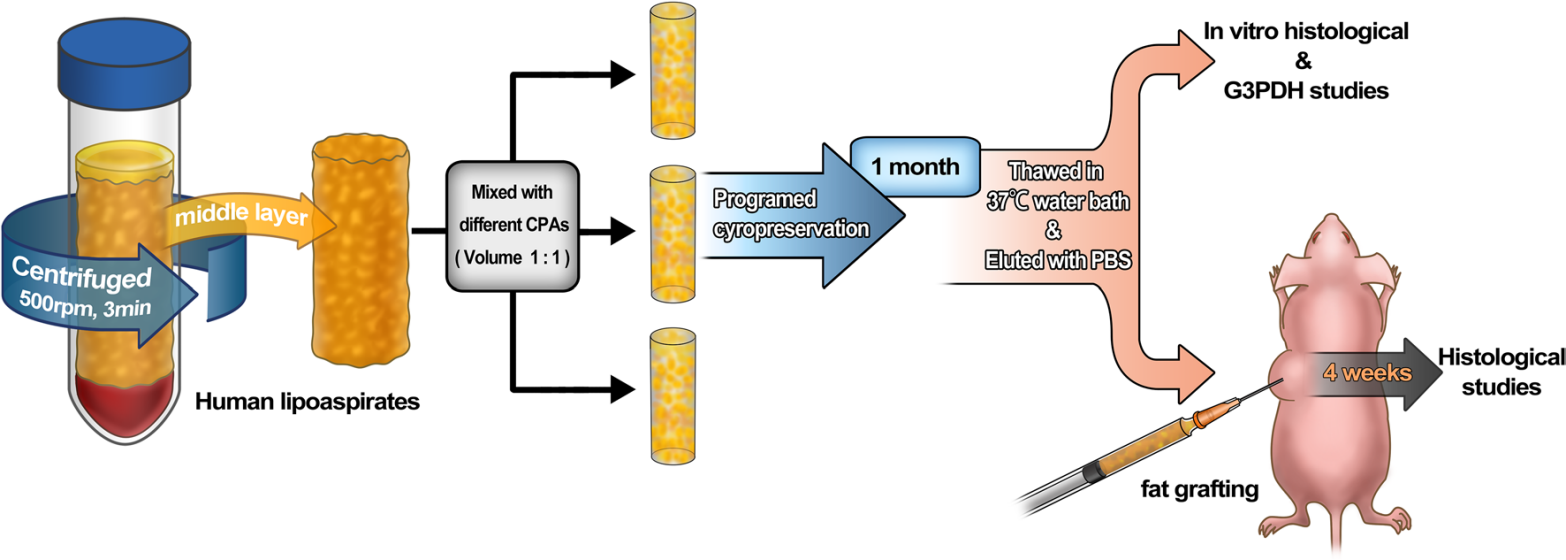
Schematic representation of the cryopreservation and experimental methods. Aspirated fat was centrifuged at 500 rpm for 3 min. The middle layer of adipose tissue was mixed with different cryoprotective agents (CPAs) at a 1:1 volume ratio. The mixtures were then cryopreserved with a programmed protocol and stored in liquid nitrogen at − 196 °C. After 1 month of cryopreservation, the tissues were thawed and eluted. In vitro and in vivo studies were then conducted

The adipose tissue was washed to remove free oil and liquid as previously reported [15]. The middle layer of pure adipose tissue was obtained and divided into 1 mL samples (Fig. 1).

### Cryopreservation of adipose tissue

Prepared adipose tissue samples were randomly divided into the following groups: (1)–(5) 60%, 70%, 80% and 90% glycerol-PBS solutions (volume/volume, V/V) and 100% pure glycerol (Bio-Rad Laboratories, Hercules, USA); (6) 0.25 mol/L trehalose(Solarbio, Beijing, China)-PBS solution as a nontoxic CPA [15]; (7) 90% fetal bovine serum (Gibco, Grand Island, USA) and 10% dimethyl sulfoxide (Sigma-Aldrich, St. Louis, USA) (FBS + DMSO) (V/V), as a positive control; and (8) no CPA (blank). Each group contained 75 mL of adipose tissue (15 mL from each volunteer, 6 volunteers in all). One milliliter of adipose aspirate was mixed with 1 mL of CPA solution at room temperature in a 5 mL cryogenic vial.

Programmed cryopreservation using a controlled-rate freezing container (Thermo Scientific, Waltham, USA) with a cooling rate of − 1 °C/min was applied in this study. Mixed samples were kept in a freezing container at − 80 °C for at least 12 h before transfer to − 196 °C liquid nitrogen as the standard protocol [7].

### Thawing and elution

After one month of cryopreservation, the samples were removed from liquid nitrogen and immediately placed in a 37 °C water bath until they were thoroughly thawed [7]. Five milliliters of PBS was added slowly to the cryopreserved tissues and mixed for 3 min to wash the tissue. The mixed samples were centrifuged at 500 rpm for 3 min to separate and remove the liquid in the lower layer. This procedure was repeated twice to thoroughly remove the CPAs.

### Fat transplantation model

All animal experiments were performed in accordance with the Animal Use Committee of the Shanghai Jiao Tong University animal guidelines and was approved by the Animal Ethics Committee of Shanghai Ninth People's Hospital. Glycerol solution (90%) and glycerol (100%) demonstrated poor cryoprotective effects in vitro and were eliminated in the in vivo studies; thus, 6 groups were studied (60% glycerol, 70% glycerol, 80% glycerol, FBS + DMSO, trehalose and blank). Twenty-four BALB/c male mice (6 weeks old, weighing 25–30 g) were randomly divided into 6 groups (4 mice/8 sides each group).

After anesthesia, prepared fat tissue (0.2 mL) was injected into one side of the nude mouse dorsum using a blunt-tip cannula. Each mouse was injected on both sides of the dorsum. Mice were killed, and the grafted fat samples were harvested and weighed after 4 weeks. Samples were divided into half for histological studies and the other half for RNA extraction.

### Biochemical activity assessment by G3PDH assay

The glyceraldehyde-3-phosphate dehydrogenase (G3PDH) activity assay kit (MAK208-1KT; Sigma-Aldrich, St. Louis, USA) was assessed the cellular activity of adipose tissues [16]. Briefly, tissue samples (10 mg) were homogenized in 200 mL of ice-cold GPDH assay buffer and then centrifuged. The supernatant was mixed with the reaction mixture, and the absorbance at 450 nm was measured with a microplate reader (BioTek, Winooski, USA) for 60 min at 37 °C. The G3PDH activity of cryopreserved tissue was calculated and compared with the fresh tissue as a control group.

### Stromal vascular fraction (SVF) isolation and viability count

The SVF was isolated from 2 mL of cryopreserved adipose tissue. Briefly, the adipose tissue was digested with collagenase for 30 min at 37 °C. Cell suspension was filtered and centrifuged and resuspended in medium. Total SVF cell numbers were measured, followed by trypan-blue staining to identify dead cells. Calcein blue acetoxymethyl (AM) /propidium iodide (PI) stain was conducted by flow cytometry to confirm the live/dead cell results (For detailed methods, see Additional file 1). Viability rate were calculated as living cells divided by total SVF cell count.

### ASCs culture and surface marker identification

Isolated SVF cells from different groups were subcultured to the 3rd passage for obtaining ASCs. The cells were cultured in low-glucose DMEM (Thermo Fisher, Waltham, USA) containing 10% FBS and 1% penicillin–streptomycin solution (GE Healthcare, Freiburg, Germany) until the confluence was 90%. ASCs were identified using cytofluorometry analysis. For detailed method of flow cytometry, see Additional file 1.

### ASCs proliferation assessment

A Cell Counting Kit-8 (CCK-8) assay was conducted to examine the ASCs proliferative [17]. Briefly, a total of 10 µL of CCK-8 reagent was added to each well on days 1, 3, 5 and 7. After incubation for 30 min at 37 °C, absorbance at 450 nm was measured using a microplate Reader (BioTek, Winooski, USA). Cell proliferation was further calculated using a standard curve.

### ASCs multi-lineage differentiation assessment

The multi-lineage differentiation capacity of the ASCs was detected as previously described [18]. Adipogenic differentiation was cultured at a concentration of 10^6^ cells/mL in the adipogenic induction medium followed by culture for 14 days. Adipogenesis was assessed by staining with 0.5% Oil red O. For osteogenic differentiation, cells were cultured in osteogenic-inducing medium for 3 weeks and stained with alizarin red S. In chondrogenic differentiation, cells were maintained in chondrogenic medium for up to 5 weeks and stained with Alcian blue. All kits were purchased from Cyagen (Santa Clara, USA) and were conducted according to manufacturer’s protocol. Images were quantified using ImageJ 1.x (National Institutes of Health, USA). For detailed method of multi-lineage differentiation assessment, see Additional file 1.

Furthermore, multi-lineage differentiation was assessed by conducting quantitative real-time PCR for specific mRNAs.

### Morphology assessment

Adipose tissues were fixed immediately in 4% paraformaldehyde, embedded in paraffin and sectioned at 6 μm for histological examination under a microscope (Nikon, Tokyo, Japan). The histological analysis was performed by H&E staining. Fibrosis was characterized by the presence of collagen fiber bundles and fibroblasts around adipocyte clusters, and vacuole was characterized as the cysts. The fibrotic and vacuole area was measured by ImageJ 1.x (National Institutes of Health, USA) [area (%) = area/total area of the same section * 100%].

For immunofluorescence study, the sections were washed and incubated at 4 °C overnight with the primary antibody against perilipin (1:200, Abcam, Cambridge, United Kingdom). The sections were then incubated with the secondary antibody incubation at 37 °C for 1 h. DAPI (Sigma-Aldrich, St. Louis, USA) was used for DNA counterstaining; 5 random views were imaged under a microscope at high magnification (Nikon, Tokyo, Japan).

### RT-PCR

mRNA extraction and purification were executed by the TRIzol method following the standard protocol [19]. Relative gene expression was calculated by the ∆∆CT method and normalized to human TATA-box binding protein (TBP) for analysis. Primers are listed in Table 1. For detailed method of RT-PCR, see Additional file 1.

**Table 1:** List and sequence of primers

	Forward primer	Reverse primer
RUNX2	AGGCAGTTCCCAAGCATTTCATCC	TGGCAGGTAGGTGTGGTAGTGAG
ALPL	GCCCTTCACTGCCATCCTGTATG	CCTGGTAGTTGTTGTGAGCATAGTCC
PPARG	TCTCCAGCATTTCTACTCCACATTACG	CAGGCTCCACTTTGATTGCACTTTG
ADIPOQ	GTGTATGGGGAAGGAGAGCGTAATG	AGTTGGTGTCATGGTAGAGAAGAAAGC
ACAN	GAGCGGCAGCACTTTGACATTTC	CACATCACTGGTGGTGGTGGATTC
SOX9	AGGAGAGCGAGGAGGACAAGTTC	TTGTTCTTGCTGGAGCCGTTGAC
IL1A	GCCAGTGAAATGATGGCTTATT	AGGAGCACTTCATCTGTTTAGG
IL6	CACTGGTCTTTTGGAGTTTGAG	GGACTTTTGTACTCATCTGCAC
TNF-α	TGCACTTTGGAGTGATCGGC	GCTACAGGCTTGTCACTCGG
CASP9	CTGCTGCGTGGTGGTCATTCTC	TCGACCGACACAGGGCATCC
TBP	CACGAACCACGGCACTGATT	TTTTCTTGCTGCCAGTCTGGAC

### Statistical analysis

All data were collected from at least three independent replications. Experimental data were analyzed using GraphPad Prism 6 (GraphPad Software, San Diego, USA). Data comparison among multiple groups was performed using one-way analysis of variance (ANOVA) and Tukey’s post hoc test. Continuous variables are shown as the mean ± SD. p < 0.05 was considered statistically significant.

## Results

### The 70% glycerol group showed the highest tissue bioactivity in the G3PDH assay

After thawing and elution, the bioactivity of cryopreserved adipose tissue was evaluated by the G3PDH assay and compared with that of freshly harvested adipose tissue. G3PDH activity was 24.76 ± 0.48 in fresh adipose tissue and 24.41 ± 0.70 in the 70% glycerol group. No significant difference was found in the G3PDH activity between these two groups (*p* > 0.99), suggesting well-preserved tissue bioactivity in the 70% glycerol group.

**Fig. 2:**
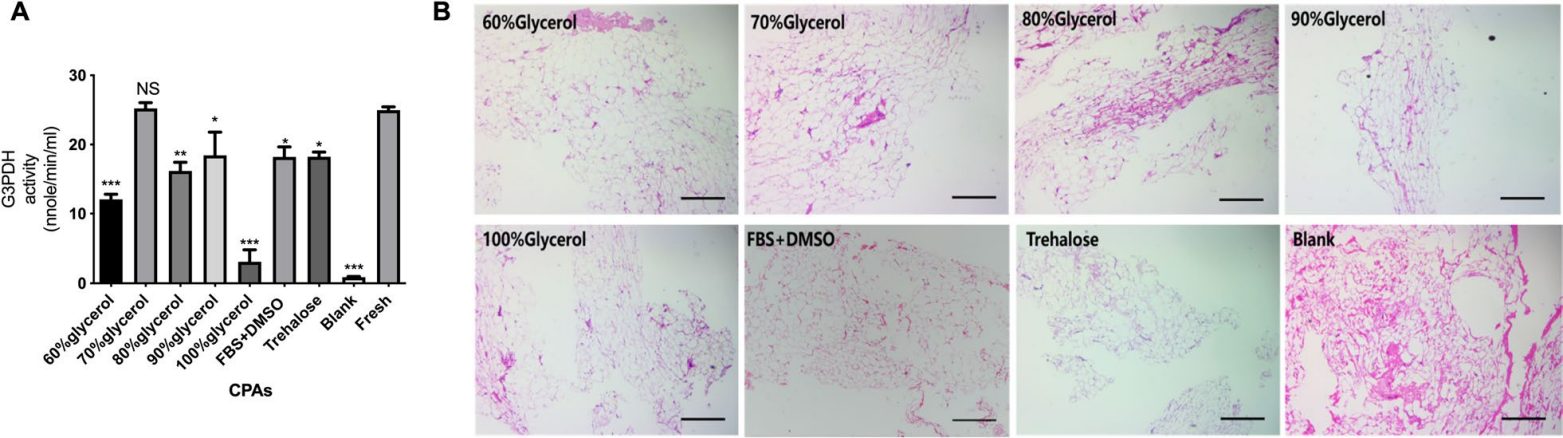
Bioactivity and morphological assay of the cryopreserved adipose tissue. **A** G3PDH assays in the CPA groups. All CPA groups were compared with fresh tissue. No significant difference was found between the 70% glycerol and fresh groups. **B** Images show HE-stained fat graft sections of 8 groups one month after fat grafting. All samples were cryopreserved at − 196 °C for 1 month. Necrosis was obvious in the blank group and the 80% glycerol group. A cyst is shown in the blank group. Scale bar: 250 µm. *p < 0.05, **p < 0.01, ***p < 0.001

The 60% glycerol (12.23 ± 0.61), 80% glycerol (15.93 ± 0.10), 90% glycerol (18.03 ± 2.47), FBS + DMSO (18.85 ± 1.49) and trehalose (18.38 ± 0.53) groups had moderately preserved G3PDH activity. These groups showed significant differences compared with the fresh group and the 70% glycerol group. The 100% glycerol group (3.49 ± 1.38) and blank group (0.92 ± 0.11) had the lowest G3PDH activity (Fig. 2A).

### The 60% and 70% glycerol, trehalose and F + D groups showed better histological features in vitro than the other groups

HE staining was performed in vitro after thawing and elution (Fig. 2B). The preserved adipose tissue showed varying degrees of tissue damage associated with freezing damage. In the CPA groups, the tissues from the DMSO + FBS, trehalose, 60% glycerol and 70% glycerol groups had a more integrated structure, while those from the 90% glycerol and 100% glycerol groups presented obvious tissue shrinkage and necrosis. Tissues from the blank group showed the most deteriorated structure.

### ASCs were better preserved in the 70% glycerol group

**Fig. 3:**
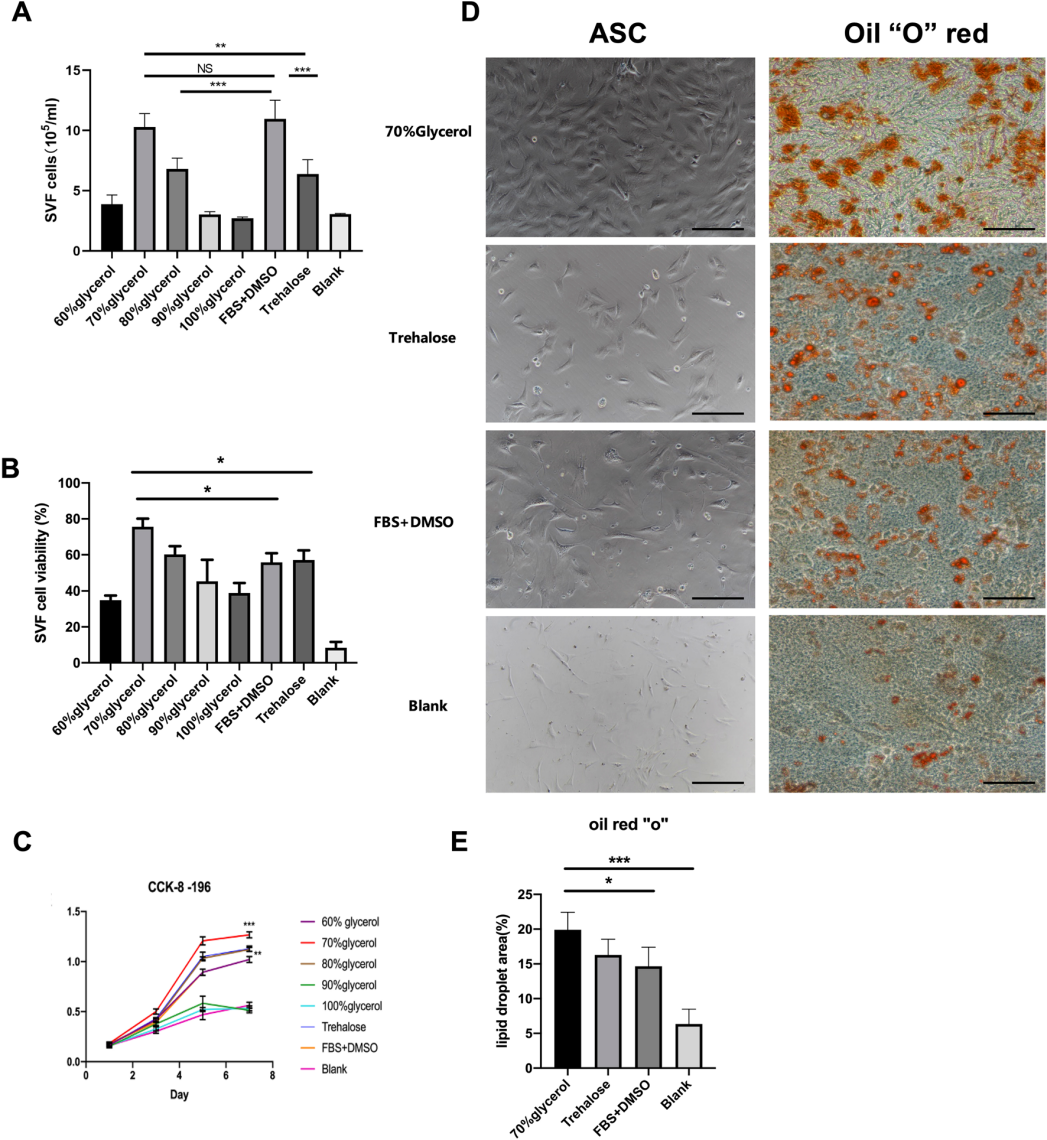
Stromal vascular fraction (SVF) cell viability and function. **A** The total number of SVF cells obtained from 2 mL of adipose tissue from different CPA groups. **B** Viability of the SVF cells. **C** CK-8 assay. **D** Left: Proliferation of ASCs. Growth density and morphology of the cells in the 70% glycerol group was better. Right: Oil red O staining showed that the 70% glycerol group had more red lipid droplets after adipogenic differentiation. **E** Quantification of lipid droplets areas after adipogenic differentiation. Scale bar: 250 µm. NS, no significance; *p < 0.05; **p < 0.01; ***p < 0.001

The total of isolated SVF cells from 2 mL of adipose tissue were counted (Fig. 3A). Cell count was the highest in the 70% glycerol group [(10.28 ± 1.13) × 10^5^ cells/mL)] and the FBS + DMSO group [11.3 ± 3.87 (× 10^5^ cells/mL)], with no significant difference. The 60%, 90% and 100% glycerol groups, as well as the blank group, showed significantly lower counts of live SVF cells.

Viability of SVF cells was studied with trypan-blue stain identifying dead cells (Fig. 3B). 70% and 80% glycerol groups had the highest SVF cell viability (72.67 ± 5.80% and 61.63 ± 3.92%), with no significant difference found (*p* = 0.147). Compared to the 70% group, the FBS + DMSO (48.37 ± 5.53%), trehalose (58.83 ± 5.61%) and 60% glycerol groups (47.76 ± 4.55%) had significantly lower SVF cell viability (p < 0.001, FBS + DMSO vs 70% glycerol, 60% glycerol vs 70% glycerol; *p* = 0.04, trehalose vs 70% glycerol). The 90% glycerol (38.6 ± 2.95%) and 100% glycerol (33.13 ± 4.96%) groups had the worst SVF cell viability (Fig. 3B). Calcein blue AM/PI stain was also conducted to verify SVF cell viability, which had shown consistent results (Additional file 1: Figs. S4, S5).

### Fat preserved in 70% glycerol showed better ASC morphology and better-preserved proliferation and multi-lineage differentiation ability than that in other groups

After sub-cultivation, the adherent ASCs presented a typical spindle-like shape. The results of the CCK-8 assay indicated that ASCs from the 70% glycerol group had a higher cell proliferation rate than those from the other cryopreserved groups (Fig. 3C, D, Additional file 1: Fig. S2A). ASCs from 70% glycerol group expressed negative CD45 and CD31, positive CD90, CD44, CD73 and CD105, indicating undifferentiated ASCs (Additional file 1: Fig. S1).

After adipogenic induction, lipid droplets areas were 19.91 ± 2.51%, 16.29 ± 2.27%, 14.66 ± 2.80%, 6.34 ± 2.15% in 70% glycerol, trehalose, FBS + DMSO and blank groups, respectively (Fig. 3D, Additional file 1: Fig. S2A). mRNA expression of PPARG and ADIPOQ was significantly higher in 70% glycerol group compared with trehalose and FBS + DMSO groups, further suggesting better adipogenic differentiation capacity (Additional file 1: Fig. S3). Blank group was eliminated from RT-PCR analysis because of too few cDNA acquired from digestion. Osteogenic and chondrogenic differentiation presented consistent results by morphology and RT-PCR analysis (Additional file 1: Figs. S2B, S3).

### Tissues from the glycerol and trehalose groups had higher retention rates after transplantation

**Fig. 4:**
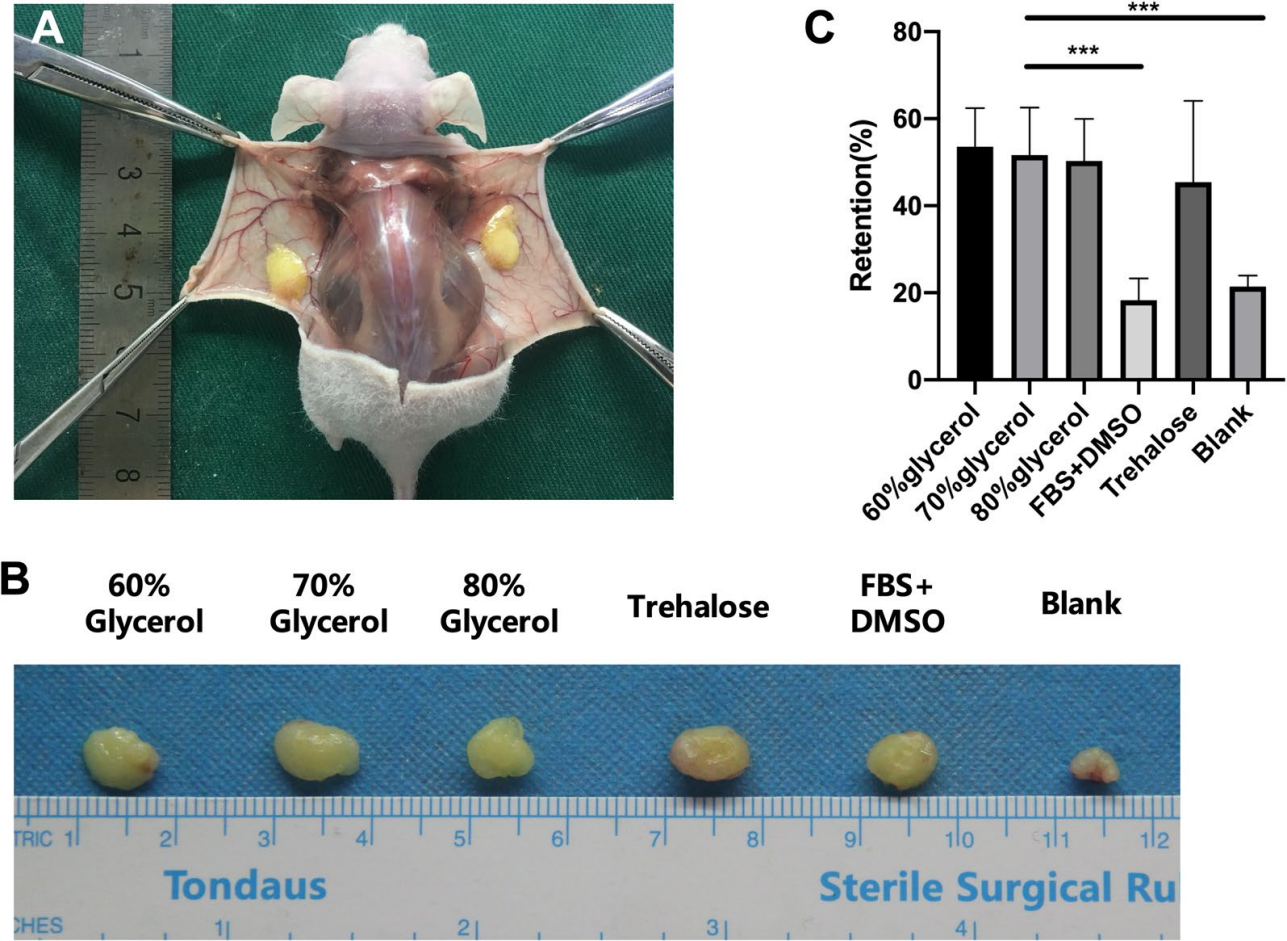
Grafted fat harvested four weeks after transplantation. **A** Fat graft analysis using nude mice. Vascularization was found to be associated with grafted fat. **B** Volume comparison of the grafted fat samples. **C** Retention rate of the CPA groups. *p < 0.05, **p < 0.01, ***p < 0.001

Four weeks after fat grafting, the transplanted tissues were harvested. Vessels were observed around the grafted adipose tissue, suggesting successful vascularization (Fig. 4A). The weight of the grafted fat was measured and calculated as volume retention (Fig. 4B). Tissues from the FBS + DMSO group had a retention rate of 19.52 ± 3.86%, while that in the blank group was 18.74 ± 4.05. These two groups had a similar retention rate (*p* > 0.99), which was significantly lower than that of the other groups. The 60% glycerol (51.87 ± 6.13), 70% glycerol (52.37% ± 7.53), 80% glycerol (47.69% ± 6.89) and trehalose groups (46.11% ± 12.26) all presented higher retention rates, with no significant difference among them (Fig. 4C).

### Adipose tissue preserved in 70% glycerol showed better histological features after transplantation than other tissues

**Fig. 5:**
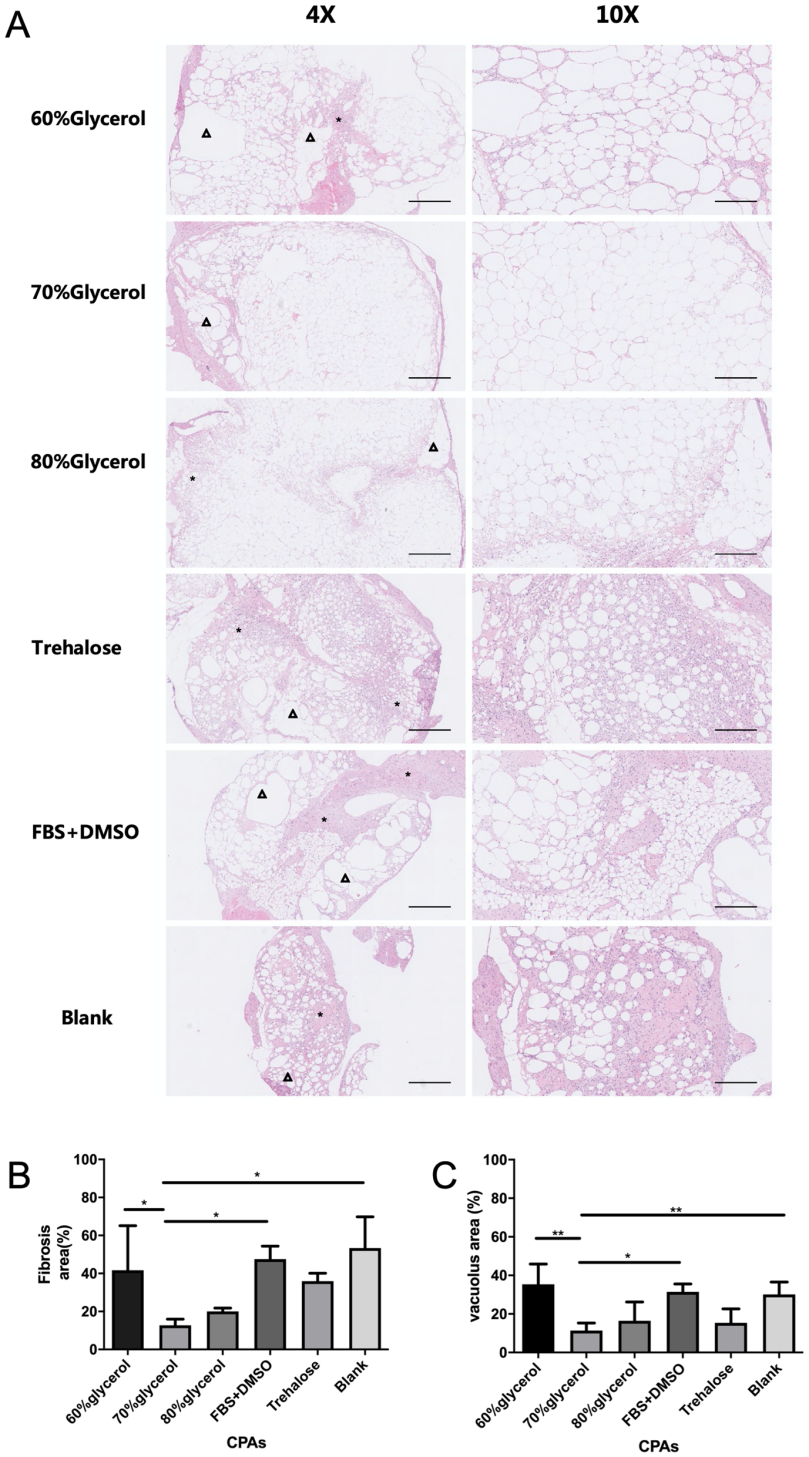
Adipose tissue histology after fat graft. **A** Representative images of HE-stained fat grafts four weeks after grafting. Preserved tissue construction and integrity were observed in the 60%, 70% and 80% glycerol groups, while the 60% glycerol group had more obvious vacuole cyst (△) formation. The trehalose group demonstrated apparent fibrosis and inflammatory infiltration (*). Fibrosis, inflammation (*) and vacuole formation (△) were detected in the FBS + DMSO group. The blank group demonstrated a disorganized structure, fibrosis and inflammatory (*) infiltration. (Scale bar: 250 µm.) **B** The fibrotic areas of grafted fat samples. **C** The vacuole areas of grafted fat samples. *p < 0.05, **p < 0.01, ***p < 0.001

The 70% glycerol group demonstrated a well-preserved tissue structure, with few areas of cysts and fibrosis. Adipocytes were plump and well organized. The blank group showed a disorganized tissue structure, in which fibrosis and vacuoles occupied most areas of the grafted tissue instead of normal adipocytes. Other groups generally demonstrated preserved structural integrity. However, among them, the 60% glycerol group showed more cysts; the 80% glycerol group and FBS + DMSO group demonstrated more fibrotic areas. The trehalose groups had a preserved general structure, but interstitial fibrosis was severe (Fig. 5A).

Histological quantification of the grafted cryopreserved tissue was performed. The fibrotic area was 12.70% ± 3.26 in the 70% group, with no significant difference compared with that of the 80% glycerol group (20.02% ± 1.77, *p* = 0.958) and the trehalose group (35.95% ± 4.17, *p* = 0.207). The fibrotic area was larger in the 60% glycerol group (41.70% ± 23.42, *p* = 0.048) and the FBS + DMSO group (47.53% ± 6.88, *p* = 0.024) than in the 70% glycerol group. The blank group had a fibrotic area up to 53.38% ± 16.39 (Fig. 5B).

The vacuole area was also calculated (Fig. 5C). The 70% glycerol group had the lowest vacuole rate (11.35% ± 3.97). The vacuole rates were moderately higher in the 80% glycerol group (16.49% ± 9.71, *p* = 0.920) and the trehalose group (15.40% ± 7.23, *p* = 0.978), with no significant difference from that in the 70% glycerol group. The other three groups, the 60% glycerol (35.35% ± 10.50), FBS + DMSO (31.49% ± 4.02) and blank groups (30.14% ± 6.41), all had significantly larger vacuole areas.

**Fig. 6:**
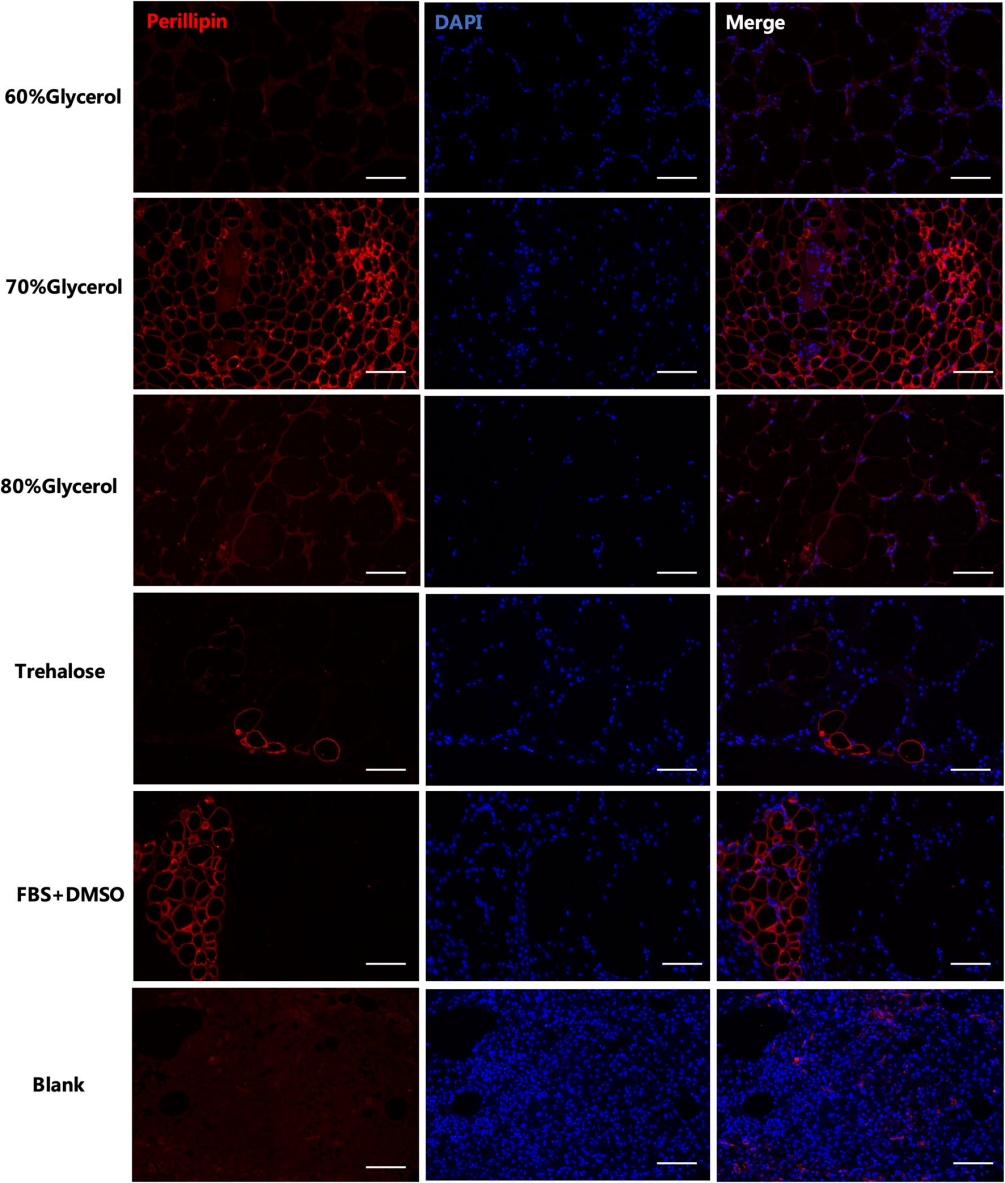
Immunofluorescence staining of grafted adipose tissue. Anti-perilipin antibody was used to mark mature adipocytes in grafted fat 4 weeks after transplantation. Red, perilipin for viable adipocytes; blue, for nuclei. Scale bars = 125 µm

Perilipin is a reliable indicator of living adipocytes [20]. The results showed that samples from the 70% glycerol group had the most preserved structure of mature adipocytes, which were round in shape and had a continuous membrane. In contrast, perilipin-positive adipocytes were lower in number or lost the oil-containing structures of the other groups (Fig. 6).

### Expression of proinflammatory cytokine genes was lower in the glycerol groups than the other groups

**Fig. 7:**
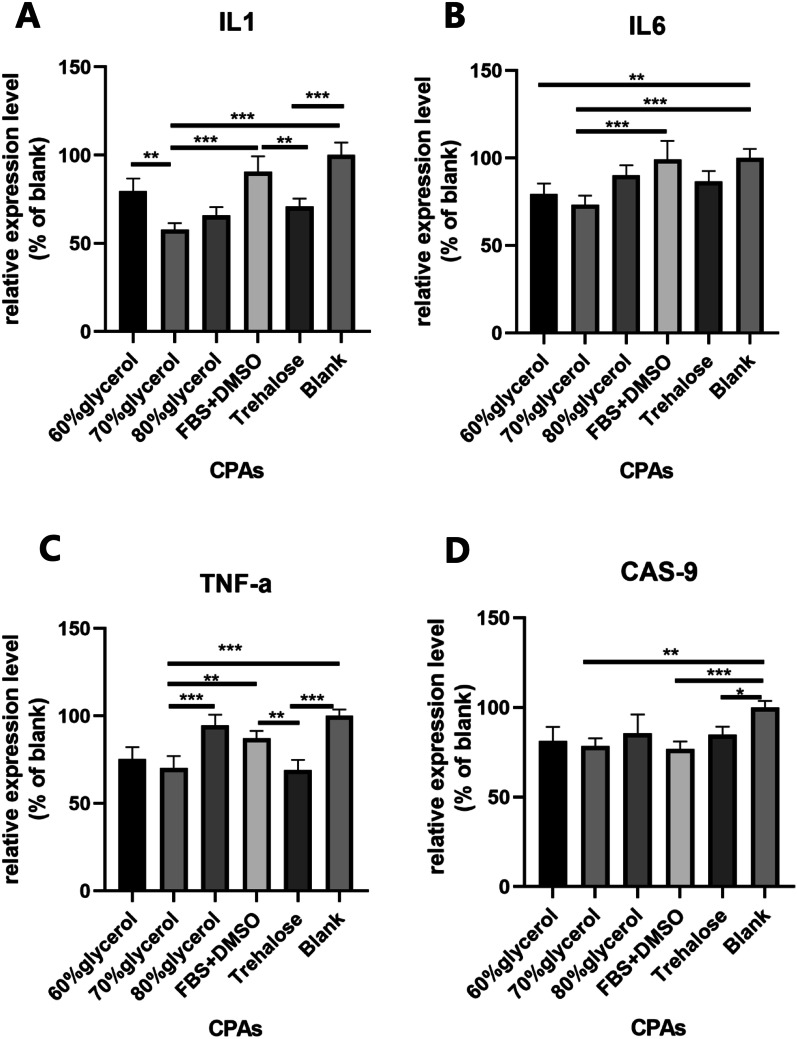
Proinflammatory and apoptotic markers in harvested adipose tissues 1 month after fat graft. The plot shows the relative expression levels of IL-1 (**A**), IL-6 (**B**), TNF-α (**C**) and Caspase-9 (**D**). *p < 0.05; **p < 0.01; ***p < 0.001

QPCR analyses evaluated the inflammatory and apoptotic activities of grafted adipose tissues. Overall, the expression of the proinflammatory cytokines IL-1, IL-6 and TNF-α was higher in the FBS + DMSO group than in the glycerol groups. Among all glycerol groups, the 70% glycerol group showed the lowest level of proinflammatory gene expression. The relative expression of IL-1 and TNF-α in the fat grafts from the 70% glycerol group was significantly lower than that in the fat grafts from the 60% and 80% glycerol groups (Fig. 7A, C). The expression of IL-1, IL-6 and TNF-α in the trehalose group was similar to that in the 70% glycerol group.

The expression of Caspase-9 was significantly higher in the blank group than in the other groups. No significant difference was found in Caspase-9 expression among the glycerol groups, trehalose group and FBS + DMSO group (Fig. 7D).

## Discussion

Proper storage of harvested adipose tissue after thawing and grafting avoids repeated liposuction procedures, thus increasing patient acceptance, reducing patient suffering, and saving time and costs [20]. Current studies regarding the efficacy and reliability of adipose tissue cryopreservation are controversial [4, 5, 21]. Lack of proper CPA is the main obstacle.

Cryopreservation of isolated cells or uniform tissues has been widely studied and applied. However, cryopreservation of composite tissues is a challenge. Different cells in the tissue may have various cryopreservation requirements [13]. Because the structure of the tissue is integrated, it is more difficult for CPAs to infiltrate the tissue thoroughly, especially for adipose tissue.

Several studies have reported that using DMSO or DMSO + FBS as CPAs was effective in protecting cell survival and retention volume of the adipose tissue compared with the absence of CPA [9, 22–25]. However, the use of DMSO and FBS has the risk of toxicity and zoonotic infection [18]. Furthermore, due to the integrity and complexity of tissue compared with isolated cells, thorough elution of DMSO may be difficult [26, 27]. Our study also found that DMSO + FBS had increased fibrosis and inflammation in cryopreserved adipose tissue.

Our study revealed that glycerol at an optimal concentration is an effective agent for adipose tissue cryopreservation. Among all concentrations tested, 70% glycerol solution appeared to be most potent in preserving tissue bioactivity. The tissue from the 70% glycerol group showed comparable activity with fresh tissue when assessed by the G3PDH assay. In vivo studies also demonstrated that the cryopreserved adipose tissue from the 70% and 80% glycerol groups had a higher retention rate and less inflammation and fibrosis after transplantation than the adipose tissue from the DMSO + FBS group. This study also showed that groups with decreased tissue activity had more in vivo fibrosis and inflammation, which is consistent with previous studies showing that oil cyst formation, chronic inflammation and progressive calcification are the results of fat necrosis [28]. Moreover, the SVF isolated from adipose tissue contains a large number of ASCs [25]. The quantity and activity of SVF cells may explain the preserved volume and structure after grafting.

The mechanism underlying the advantages of glycerol in adipose tissue preservation may be as follows: compared with other tissues, adipose tissue has unique characteristics because it is mainly comprised of adipocytes, which contain large amounts of triglycerides. The chemical similarity of glycerol and triglyceride may help cryopreservation. Studies have shown that glycerol, as the backbone of triglycerides stored in adipocytes, is transported outward through glycerol transporters [14]. With an increased concentration of glycerol in the extracellular matrix, glycerol efflux might be inhibited, thus better preserving the structure and bioactivity of adipocytes [29]. Moreover, glycerol is nontoxic and generally nonallergenic to cells and tissues, characteristics that make it suitable for clinical use [13].

We evaluated the cryopreserved tissue morphology, bioactivity and immunohistochemical staining. Histological observation was critical to determine the structural features of the tissue. Immunofluorescence provided further information on cell viability and plasma membrane continuity. Bioactivity in our study was mainly studied by G3PDH assay. The G3PDH assay was conducted on the entire tissue without digestion, and assessed cellular function of fatty tissues due to adipocyte specificity that previously reported [30]. Our results showed that when tissue bioactivity deteriorated, increased levels of fibrosis and inflammation were observed after transplantation. Although the volume retention rate did not decrease sharply, the integrity of the adipose tissue structure was destroyed, with a large area of oil cysts and fibrosis [20].

In this study, we cryopreserved adipose tissue for 1 month. Previous studies on adipose tissue cryopreservation used various freezing durations, from days to 2 months [2, 6, 7, 9, 20, 31]. Whether cryopreservation duration would affect the viability of cells and tissues remains controversial. While Ntai et al. reported that there was no difference in the viability of pluripotent stem cells cryopreserved for 7 days and for 30 days [32], Guttridge et al. indicated that cryopreservation duration may affect the viability of cryopreserved blood cells [33]. Moreover, there are no present study concerning the effect of storage time on complex tissues, such as adipose tissue. Thus, the impact of storage duration on adipose tissue cryopreservation may be required in future research. Another limitation is that although glycerol is nontoxic and preclinical research has shown good efficacy, further clinical trials are required to evaluate its clinical efficiency.

## Conclusions

Our study demonstrated that glycerol solutions can serve as CPAs for long-term cryopreservation of adipose tissue. Among all concentrations studied, 70% glycerol solution was superior at preserving adipose tissue bioactivity, reducing the retention rate, and preventing tissue fibrosis and inflammation in cryopreserved adipose tissue. Furthermore, glycerol is a nontoxic and nonimmunogenic agent and thus is safer and more compatible with clinical use than other CPAs. Future studies are required to further prove the safety and optimize the reliability of adipose tissue cryopreservation using this method.

## Supplementary Information

**Additional file 1.** Supplementary materials, including supplemental methods and the supplemental figures S1–S5. The supplemental methods contain the protocols of following experiments: Live/dead cell stain and flow cytometry, RT-PCR, flow cytometry and surface marker identification, ASCs multi-lineage differentiation assessment, quantify lipid droplets area, and immunofluorescence study.

## Data Availability

The datasets used and/or analyzed during the current study are available from the corresponding author on reasonable request.

## Abbreviations

ASC: Adipose-derived stem cell
CCK8: Cell Counting Kit-8
CPA: Cryoprotective agent
DMSO: Dimethyl sulfoxide
FBS: Fetal bovine serum
G3PDH: Glyceraldehyde-3-phosphate dehydrogenase
