# Supplementary material for: The effect of glycerol as a cryoprotective agent in the cryopreservation of adipose tissue
Source: Stem Cell Res Ther. 2022 Apr 8;13:152. doi: 10.1186/s13287-022-02817-z (PMC8994386; doi:10.1186/s13287-022-02817-z)
Supplement: Supplementary file 1 — Additional file 1. Supplementary materials, including supplemental methods and the supplemental figures S1–S5. The supplemental methods contain the protocols of following experiments: Live/dead cell stain and flow cytometry, RT-PCR, flow cytometry and surface marker identification, ASCs multi-lineage differentiation assessment, quantify lipid droplets area, and immunofluorescence study. [file 13287_2022_2817_MOESM1_ESM.docx]

**SUPPLEMENTARY MATERIALS**

1. **Supplemental Method**
   1. **Live/dead cell stain and flow cytometry**

Calcein blue acetoxymethyl (AM) is membrane-permeable live-cell labeling dye. Upon entering the cell, intracellular esterases cleave the acetoxymethyl ester group, yielding the membrane-impermeable calcein blue fluorescent dye. Propidium iodide (PI) is a membrane impermeable molecule (*668 Da) that is capable of binding to DNA and RNA only upon the loss of cellular membrane integrity in dying, dead, and necrotic cells. Therefore, the calcein blue AM/ PI method was utilized to further examine SVF cell viability in our study.

All experiments were conducted according to manufactuer’s protocol. Briefly, 1×10^5^ SVF cells (100ul) were incubated with 10 umol/L Calcein Blue-AM Dye (ThermoFisher, Waltham, USA) for 15 min and 10ul PI (ThermoFisher, Waltham, USA) for 5 min at room temperature in the dark. The suspension was analyzed in the flow cytometer and gated for biparametric histograms PI yellow fluorescence(585nm) versus Calcein blue AM violet fluorescence (450nm). Experiment was conducted in triplicate.

- 1. **RT-PCR**

mRNA extraction and purification were executed by the TRIzol method. RNA integrity and quantity were assessed with a NanoDrop 8000 Microvolume UV-Vis spectrophotometer (Thermo Fisher Scientific, Waltham, USA). For quantitative real-time PCR analysis, 500–2000 ng of total RNA was reverse transcribed using the cDNA Reverse Transcription kit (PrimeScript RT Master Mix Perfect Real Time, Takara Bio, Kusatsu, Japan), SYBR Green PCR master mix and 300 nM primers on an Applied Biosystem StepOne instrument (Thermo Fisher Scientific, Waltham, USA). Relative gene expression was calculated by the ∆∆CT method and normalized to housekeeping GAPDH gene for human analysis. Primers are listed in Table 1. All mRNA expression analyses were performed in a blinded manner.

- 1. **Flow cytometry and surface marker identification**

ASCs were identified using cytofluorometry analysis. ASCs were incubated with monoclonal antibodies against APC conjugated CD45, B525-FITC conjugated CD31, PE conjugated CD90, Y585-PE conjugated CD44, APC conjugated CD73, B690-PC5.5 and conjugated CD105 (all antibodies purchased from BD Biosciences, USA).

ASC cells were resuspended using 100ul staining PBS buffer containing 3% FBS. After that, cells were counted and diluted to 1×106 cell/ml. 100ul cell suspension (1×10^5^ cells) was transferred to a 1.5ml EP tube. Then cell suspension was mixed with antibodies according to manufacturer’s guide for 40 minutes on ice, avoided from light. Then 100ul flow cytometry buffer was added to each EP tube. After centrifuge for 5min at 1200rpm (4 ℃), supernatant was abandoned, and cells were fixed with 100ul paraformaldehyde for 30 minutes and then resuspended with 100ul flow cytometry buffer. Samples were examined using a FACS Aria-flow cytometer (Becton-Dickinson, San Jose, CA, USA). Mouse IgG1 isotype control APC conjugated, or mouse IgG1 isotype control FITC conjugated, or mouse IgG1 isotype control PerCP conjugate, or mouse IgG1 isotype control PE conjugated were used as isotype matched controls.

- 1. **ASCs multi-lineage differentiation assessment**

Adipogenic differentiation was cultured at a concentration of 10^6^ cells/ml in the adipogenic induction medium followed by culture for 14 days, with adipogenic differentiation medium changed twice weekly. Adipogenesis was assessed by staining with 0.5% Oil red O solution at room temperature for 60 minutes to detect intracellular lipids. Images were analyzed from 5 random views and lipid droplets area were quantified using ImageJ 1.x (National Institutes of Health, USA).

For osteogenesis, MSCs were seeded on gelatine-coated NuncTM 24-well plates (Thermo Fisher, USA) at a density of 1.5 × 10 cells/cm . After 24 h, differentiation was induced. Cells were regularly checked for morphology, and the medium was exchanged every 3–4 days. Osteogenic differentiation was conducted for 3weeks before staining was performed. 0.1% alizarin red was used and staining was performed for 20 mins. Chondrogenic differentiation was performed using the micromass culture technique; cells were maintained in chondrogenic medium for up to 5 weeks and stained with Alcian blue.

Images were analyzed from 5 random views and stained area were quantified using ImageJ 1.x (National Institutes of Health, USA).

- 1. **Quantify Lipid Droplets Area**

Image analysis for quantification of lipid droplets area was performed with the open-source

software Fiji. For any mathematical operation during the analysis steps, images were converted into a 32-bit floating point format to avoid pixel saturation. Region-of-interest (ROI) selection was conducted with a color threshold auto measurement method. Analyzed parameters included area and area fraction. Images were analyzed from 5 random views.

- 1. **Immunofluorescence study**

The sections were deparaffinized, hydrated, and rinsed with distilled water. Then, the sections were consumed in a peroxidase-blocking solution, followed by heat-induced epitope retrieval. The sections were washed and incubated at 4°C overnight with the primary antibody against perilipin (1:200, Abcam, Cambridge, United Kingdom). After incubation, the sections were washed twice prior to secondary antibody incubation at 37°C for 1 hour. DAPI (Sigma-Aldrich, St. Louis, USA) was used for DNA counterstaining. The sections were mounted on slides. 5 random views were imaged under a microscope at high magnification (Nikon, Tokyo, Japan).

1. **Supplemental Figure**


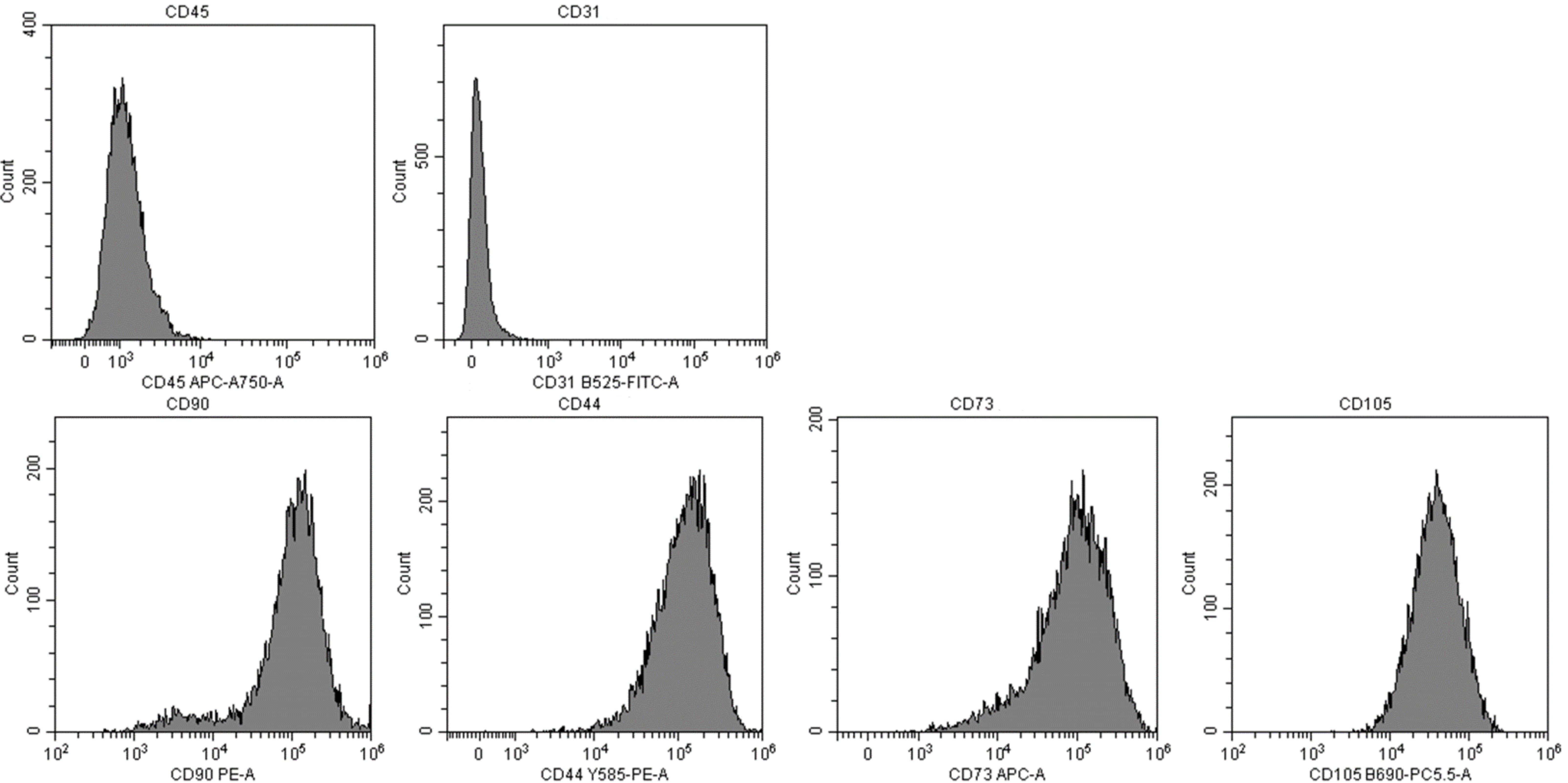


**Figure S1.** **Expression of the surface markers of isolated SVF cells from cryopreserved adipose tissue with different CPAs.**


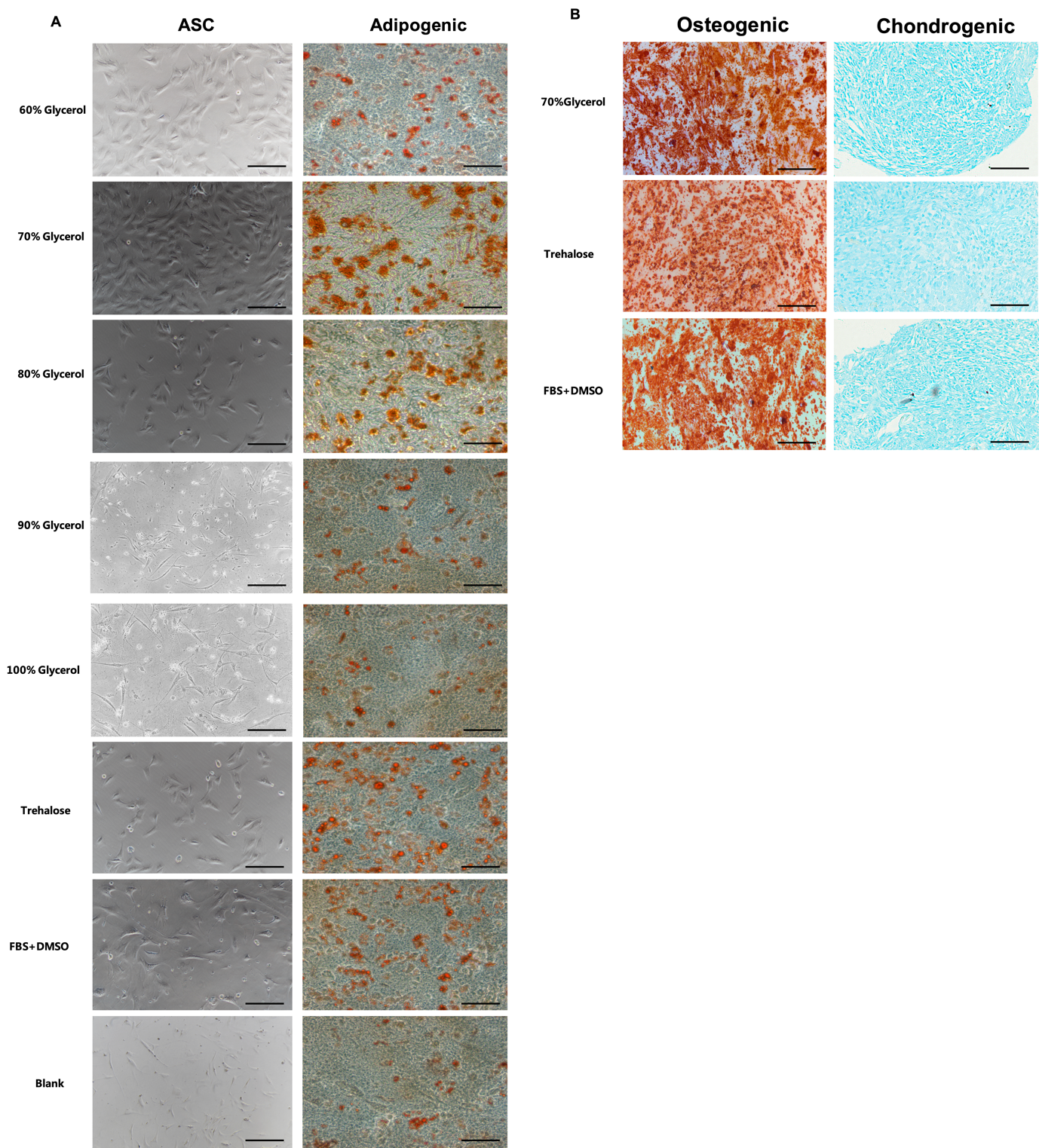


**Figure S2. Proliferation and multi-lineage differentiation of ASCs isolated from cryopreserved adipose tissue**

(A) Left: Microscopic image of ASCs proliferation. Right: Microscopic image of adipogenic differentiation. Red staining indicates Oil Red O-stained lipid droplets. (B) Microscopic image of osteogenic (left) and chondrogenic (right) differentiation of ASCs.


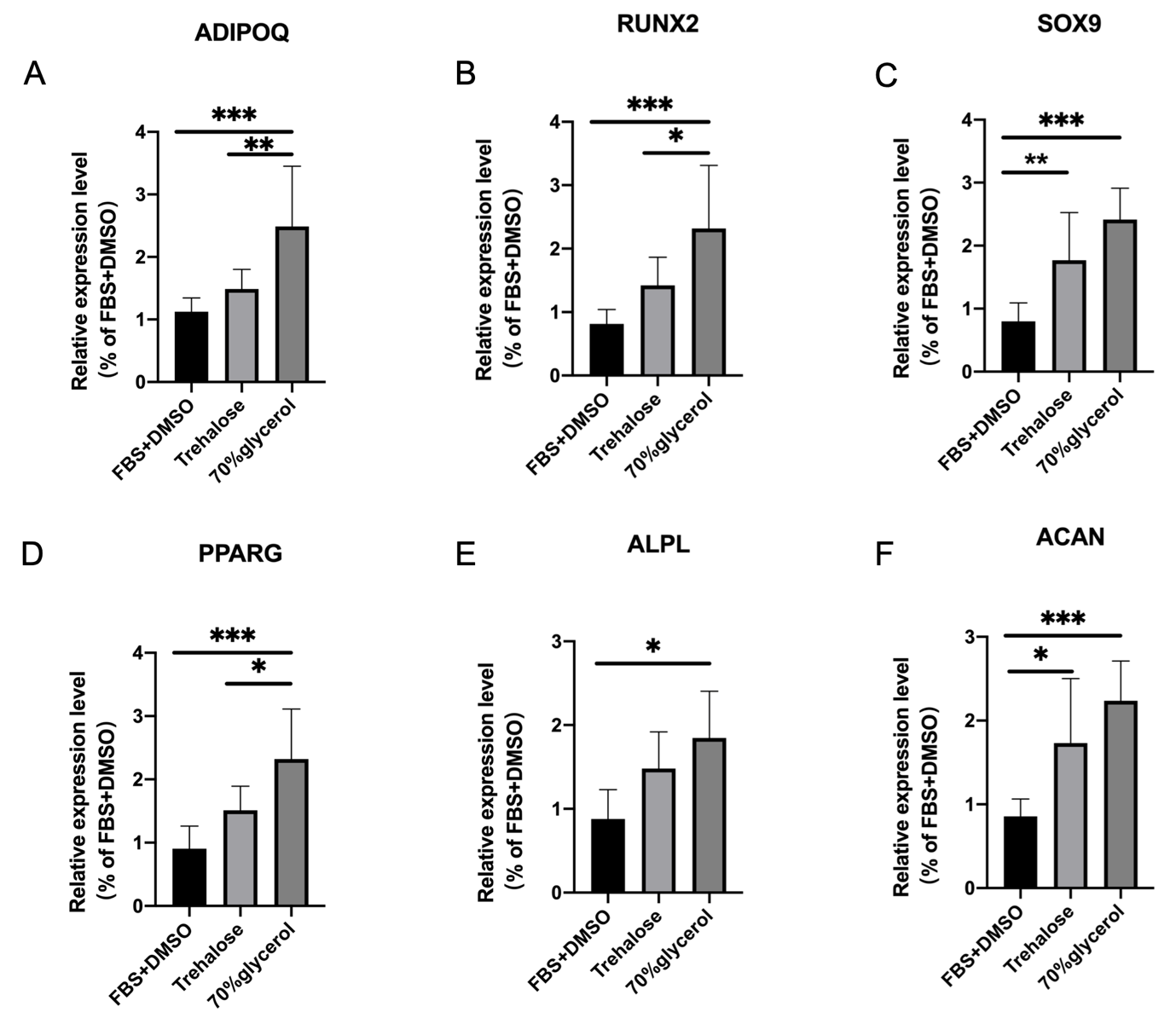


**Figure S3. Adipocyte markers after adipogenic differentiation of ASCs isolated from cryopreserved adipose tissue.** *p < 0.05, **p < 0.01, ***p < 0.001


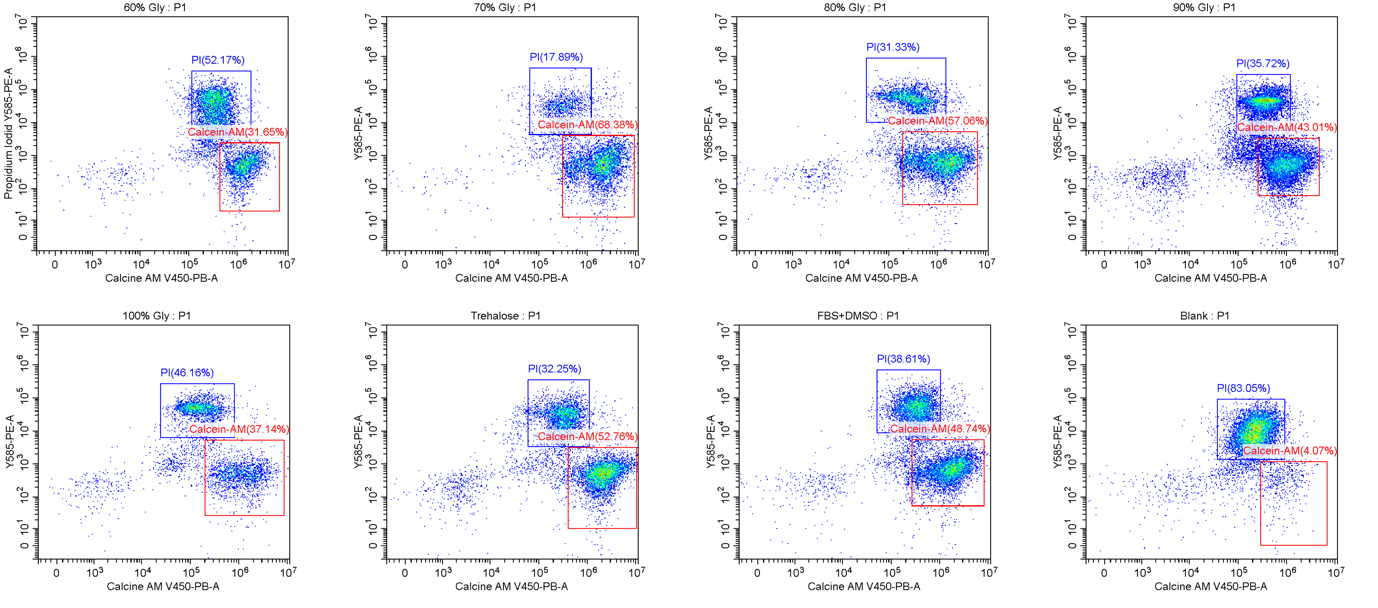


**Figure S4. Flow cytometry for living/dead SVF cells using calcine blue AM/propidum iodide stain.**

**
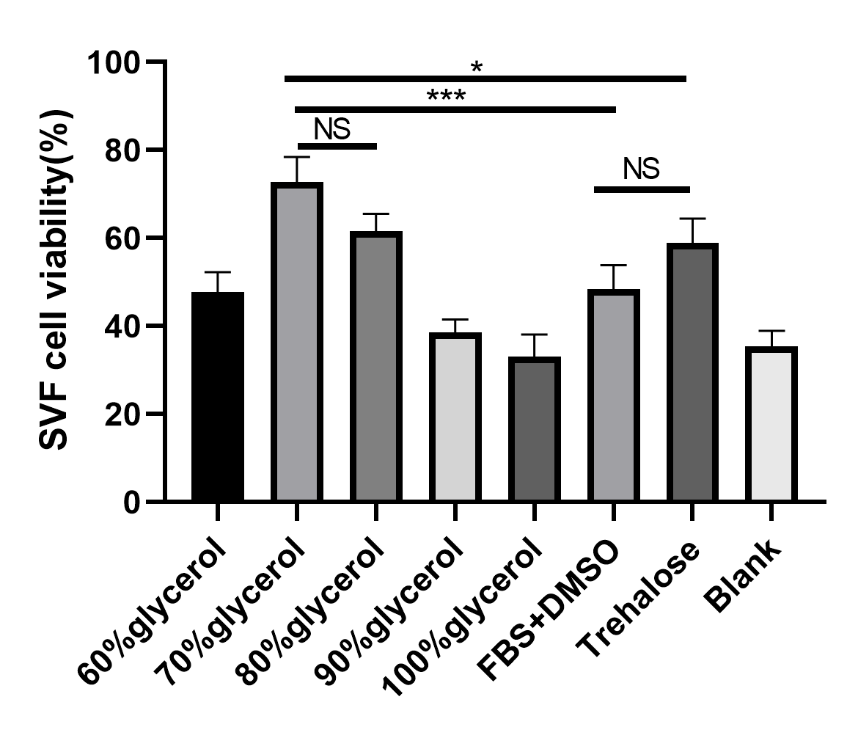
**

**Figure S5. SVF viability calculated from Trypan blue stain.**

70% and 80% glycerol groups had the highest SVF cell viability (72.67 ± 5.80% and 61.63 ± 3.92%), with no significant difference found (p=0.147). Compared to the 70% group, the FBS+DMSO (48.37 ± 5.53), trehalose (58.83 ± 5.61) and 60% glycerol groups (47.76 ± 4.55) had significantly lower SVF cell viability (p<0.001, FBS+DMSO vs 70% glycerol, 60% glycerol vs 70% glycerol; p=0.04, trehalose vs 70% glycerol). The 90% glycerol (38.6 ± 2.95) and 100% glycerol (33.13 ± 4.96) groups had the worst SVF cell viability . These results shown consistent trends with previous Calcein blue AM/PI stain results (Figure 3B). *p < 0.05
